# 11-{[2-(3-Fluoro­phen­yl)eth­yl](meth­yl)amino}­penta­cyclo­[5.4.0.0^2,6^.0^3,10^.0^5,9^]undecan-8-one

**DOI:** 10.1107/S1600536810038523

**Published:** 2010-09-30

**Authors:** Samuel D. Banister, Jack K. Clegg, Raphy Hanani, Michael Kassiou

**Affiliations:** a School of Chemistry, F11, The University of Sydney, New South Wales 2006, Australia; b Department of Chemistry, University of Cambridge, Lensfield Rd, Cambridge CB2 1EW, England; c Brain and Mind Research Institute, Sydney, New South Wales 2050, Australia, Discipline of Medical Radiation Sciences, The University of Sydney, New South Wales 2006, Australia

## Abstract

In the title compound, C_20_H_22_FNO, the distances close to the carbonyl and amine are: N—O = 3.232 (4) Å and N—C = 2.666 (5) Å. The crystal packing is unremarkable.

## Related literature

For *in vitro* σ-receptor affinity of tris­homocubane derivatives related to the title compound, see: Nguyen *et al.* (1996 ▶); Liu *et al.* (1999 ▶). For *in vivo* pharmacology of related tris­homocubanes, see: Liu *et al.* (2001 ▶, 2007 ▶). For rationalization of observed structure–affinity relationships of tris­homocubanes at σ-receptors using mol­ecular modeling, see: Banister *et al.* (2010 ▶). For X-ray crystallographic studies of biologically active tris­homocubanes related to the title compound, see: Hambley *et al.* (2000 ▶).
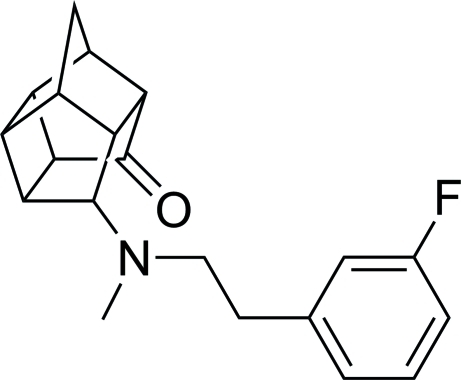


## Experimental

### 

#### Crystal data

C_20_H_22_FNO
*M* *_r_* = 311.39
Monoclinic, 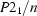

*a* = 10.5450 (18) Å
*b* = 10.980 (2) Å
*c* = 13.822 (3) Å
β = 95.214 (8)°
*V* = 1593.8 (5) Å^3^
*Z* = 4
Mo *K*α radiation
μ = 0.09 mm^−1^
*T* = 150 K
0.25 × 0.20 × 0.15 mm

#### Data collection

Bruker–Nonius APEXII FR591 diffractometer
Absorption correction: multi-scan (*SADABS*; Sheldrick, 1999 ▶) *T*_min_ = 0.684, *T*_max_ = 0.746
17270 measured reflections
2773 independent reflections
1566 reflections with *I* > 2σ(*I*)
*R*_int_ = 0.087

#### Refinement

*R*[*F* ^2^ > 2σ(*F* ^2^)] = 0.087
*wR*(*F* ^2^) = 0.271
*S* = 1.14
2773 reflections
209 parameters
H-atom parameters constrained
Δρ_max_ = 0.69 e Å^−3^
Δρ_min_ = −0.48 e Å^−3^

### 

Data collection: *APEX2* (Bruker–Nonius, 2003 ▶); cell refinement: *SAINT* (Bruker–Nonius, 2003); data reduction: *SAINT* and *XPREP* (Bruker–Nonius, 2003 ▶); program(s) used to solve structure: *SIR97* (Altomare *et al.*, 1999 ▶); program(s) used to refine structure: *SHELXL97* (Sheldrick, 2008 ▶); molecular graphics: *ORTEP-3* (Farrugia, 1997 ▶), *WinGX* (Farrugia, 1999 ▶) and *POV-RAY* (Cason, 2002 ▶); software used to prepare material for publication: *enCIFer* (Allen *et al.*, 2004 ▶).

## Supplementary Material

Crystal structure: contains datablocks global, I. DOI: 10.1107/S1600536810038523/rk2233sup1.cif

Structure factors: contains datablocks I. DOI: 10.1107/S1600536810038523/rk2233Isup2.hkl

Additional supplementary materials:  crystallographic information; 3D view; checkCIF report

## Figures and Tables

**Fig. 1.**
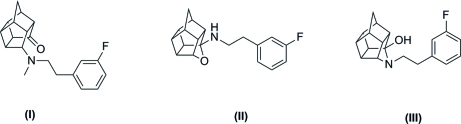
Chemical structures of I, II and III.

**Fig. 2.**
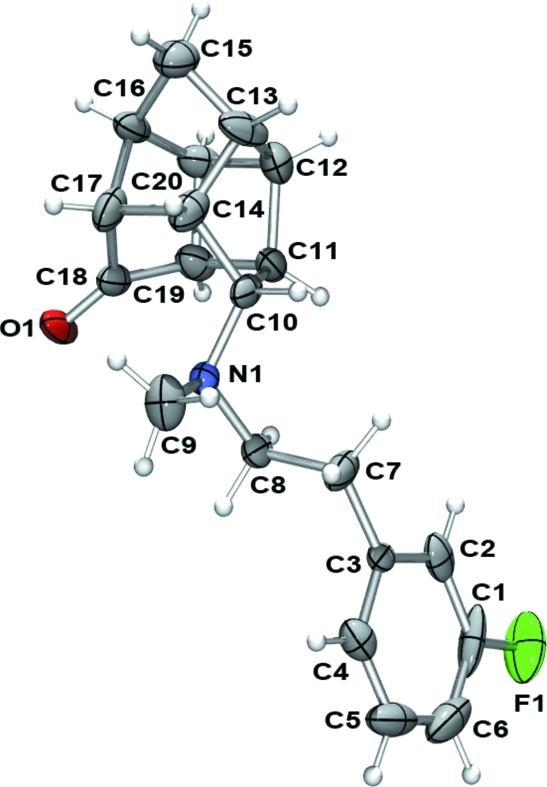
221ORTEP representation of III with atom numbering scheme. Displacement ellipsoids are shown at 50% probability level. H atoms are presented as a small spheres of arbitrary radius.

## supplementary crystallographic information

### Comment

Trishomocubanes have been shown to have *in vitro*σ-receptor affinity and selectivity [Nguyen *et al.*, (1996); Liu *et al.*, (1999)] and a number of their crystal structures have been reported [Hambley *et al.*, (2000)]. Several trishomocubane derivatives synthesized in our laboratory were reported to possess anti-cocaine activity *in vivo* [Liu *et al.*, (2001), (Liu *et al.*, (2007)]. The importance of the nature ofthe hemiaminal bridge of *N*-(2-(3-fluorophenyl)ethyl)-4-azahexacyclo [5.4.1.0^2,6^.0^3,10^.0^5,9^.0^8,11^]dodecan-3-ol I *(Fig. 1) to σ-receptor binding was demonstrated by the reduced affinity, and off-target activity, of the corresponding hemiaminal ether, *N*-(2-(3-fluorophenyl)ethyl)-3-amino-4-oxapentacyclo [5.4.1.0^2,6^.0^3,10^.0^5,9^.0^8,11^]dodecane II [Banister *et al.*, (2010)] (Fig. 1). In our ongoing efforts to elucidate the nature of σ-receptor binding we have synthesized the title compound III  (Fig. 1) as a methyl homologue of I, representing a "locked" form of the non-transannular, aminoketone tautomer of the latter. A molecular and crystal structures were obtained to unambiguously confirm the structure of III (Fig. 2), and to identify key interatomic distances, for use in modeling studies. Important distances are those close to the carbonyl and amine, including: N1–O1 = 3.232 (4)Å; N1–C18 = 2.666 (5)Å; O1–C9 = 3.943 (5)Å; C9–C18 = 3.574 (6)Å.

### Experimental

A solution of *N*-(2-(3-fluorophenyl)ethyl)-4-azahexacyclo [5.4.1.0^2,6^.0^3,10^.0^5,9^.0^8,11^]dodecan-3-ol (942 mg, 3.17 mmol) and 37% aqueous formaldehyde (285 µ*L*, 3.80 mmol, 1.2 equiv.) in ClCH_2_CH_2_Cl (30 ml) was treated with NaBH(O*Ac*)_3_ (3.359 g, 15.85 mmol, 5 equiv.) and the mixture stirred for 18 h. The reaction was quenched with 1 *M* aqueous NaOH (30 ml), and the layers separated. The aqueous layer was extracted with CH_2_Cl_2_ (3 × 15 ml) and the combined organic layers were washed with brine (25 ml), dried (Na_2_SO_4_) and the solvent evaporated. Purification was achieved using column chromatography on silica eluting with CHCl_3_-*Me*OH-conc. aq. NH_4_OH (90:9:1) to give *N*-(2-(3-fluorophenyl)ethyl)-*N*-methyl-11-aminopentacyclo [5.4.0.0^2,6^.0^3,10^.0^5,9^]undecan-8-one III as colourless crystals (902 mg, 91%): m. pt. 363-364.5 K; *R*_f_ 0.43 (90:9:1 *v*/*v*/v CHCl_3_:*Me*OH: conc. aq. NH_4_OH); IR (thin film) cm^-1^; 2970, 2861, 1721 (C═O), 1582, 1484, 1426, 1343, 1229, 1141, 1059, 981, 939, 906, 791; ^1^H NMR (400 MHz, CDCl_3_); δ 7.25-7.19 (1*H*, m, *Ar*H), 6.93 (1*H*, d, J = 7.9 Hz, *Ar*H), 6.89-6.85 (2*H*, m, *Ar*H), 3.02-2.97 (1*H*, m, CH), 2.87-2.67 (7*H*, m, CH), 2.66-2.62 (2*H*, m, CH), 2.50 (1*H*, t, J = 4.2 Hz, CH), 2.47-2.43 (1*H*, m, CH), 2.35-2.31 (1*H*, m, CH), 2.30 (3*H*, s, CH_3_), 1.86 (1*H*, d, J = 10.8 Hz, CHCH_2_CH), 1.48 (1*H*, d, J = 10.8 Hz, CHCH_2_CH); ^13^C NMR (100.6 MHz, CDCl_3_); δ 213.1 (C═O), 163.0 (3'-C, ^1^J_C–F_ = 245.3 Hz), 143.4 (1'-C, ^3^J_C–F_ = 7.4 Hz), 129.9 (5'-C, ^3^J_C–F_ = 8.3 Hz), 124.5 (6'-C, ^4^J_C–F_ = 2.6 Hz), 115.6 (2'-C, ^2^J_C–F_ = 20.8 Hz), 112.9 (4'-C, ^2^J_C–F_ = 21.1 Hz), 64.6 (CH), 57.1 (CH_2_), 51.6 (CH), 50.1 (CH), 46.4 (CH), 42.1 (CH), 41.6 (CH), 41.4 (CH), 40.8 (CH), 40.2 (CH), 38.5 (CH_2_), 37.2 (CH), 31.7 (CH_2_); m/*z* (+ESI) 312.13 ([*M* + H]^+^, 100); Anal. (C_20_H_22_NOF): calc, C 77.14, H 7.12, N 4.50; found, C 76.90, H 7.19, N 4.55. Crystals suitable for X-ray diffraction were grown by the slow evaporation of a hexane solution.

### Refinement

C bound H atoms were included in idealized positions and refined using a riding-model approximation with aromatic C–H bond lengths fixed at 0.95Å and aliphatic bond lengths at 1.00Å, 0.99Å and 0.98Å for methine, methylene and methyl H atoms respectively. *U*_iso_(H) values were fixed at 1.2*U*_eq_ of the parent C atoms, except for the methyl protons, which were fixed at 1.5*U*_eq_(C). The highest residual peak is 0.69 eÅ^-3^ and is located 1.17Å from C12 with the deepest hole -0.47 eÅ^-3^ 1.07Å from F1.

### Crystal data

**Table:**

C_20_H_22_FNO	*F*(000) = 664
*M**_r_* = 311.39	*D*_x_ = 1.298 Mg m^−^^3^
Monoclinic, *P*2_1_/*n*	Melting point: 363.5 K
Hall symbol: -P 2yn	Mo *K*α radiation, λ = 0.71073 Å
*a* = 10.5450 (18) Å	Cell parameters from 2297 reflections
*b* = 10.980 (2) Å	θ = 2.7–23.1°
*c* = 13.822 (3) Å	µ = 0.09 mm^−^^1^
β = 95.214 (8)°	*T* = 150 K
*V* = 1593.8 (5) Å^3^	Block, colourless
*Z* = 4	0.25 × 0.20 × 0.15 mm

### Data collection

**Table:**

Bruker–Nonius APEXII FR591 diffractometer	2773 independent reflections
Radiation source: rotating anode	1566 reflections with *I* > 2σ(*I*)
graphite	*R*_int_ = 0.087
ω and φ scans	θ_max_ = 25.0°, θ_min_ = 3.0°
Absorption correction: multi-scan (*SADABS*; Sheldrick, 1999)	*h* = −12→12
*T*_min_ = 0.684, *T*_max_ = 0.746	*k* = −13→12
17270 measured reflections	*l* = −16→16

### Refinement

**Table:**

Refinement on *F*^2^	Primary atom site location: structure-invariant direct methods
Least-squares matrix: full	Secondary atom site location: difference Fourier map
*R*[*F*^2^ > 2σ(*F*^2^)] = 0.087	Hydrogen site location: inferred from neighbouring sites
*wR*(*F*^2^) = 0.271	H-atom parameters constrained
*S* = 1.14	*w* = 1/[σ^2^(*F*_o_^2^) + (0.1415*P*)^2^ + 0.2766*P*] where *P* = (*F*_o_^2^ + 2*F*_c_^2^)/3
2773 reflections	(Δ/σ)_max_ < 0.001
209 parameters	Δρ_max_ = 0.69 e Å^−^^3^
0 restraints	Δρ_min_ = −0.47 e Å^−^^3^

### Special details

**Table:**

Experimental. The crystal was coated in Exxon Paratone N hydrocarbon oil and mounted on a thin mohair fibre attached to a copper pin. Upon mounting on the diffractometer, the crystal was quenched to 150 K under a cold nitrogen gas stream supplied by an Oxford Cryosystems Cryostream and data were collected at this temperature.
Geometry. All s.u.'s (except the s.u. in the dihedral angle between two l.s. planes) are estimated using the full covariance matrix. The cell s.u.'s are taken into account individually in the estimation of s.u.'s in distances, angles and torsion angles; correlations between s.u.'s in cell parameters are only used when they are defined by crystal symmetry. An approximate (isotropic) treatment of cell s.u.'s is used for estimating s.u.'s involving l.s. planes.
Refinement. Refinement of *F*^2^ against ALL reflections. The weighted *R*-factor *wR* and goodness of fit *S* are based on *F*^2^, conventional *R*-factors *R* are based on *F*, with *F* set to zero for negative *F*^2^. The threshold expression of *F*^2^ > σ(*F*^2^) is used only for calculating *R*-factors(gt) *etc*. and is not relevant to the choice of reflections for refinement. *R*-factors based on *F*^2^ are statistically about twice as large as those based on *F*, and *R*-factors based on ALL data will be even larger.

### Fractional atomic coordinates and isotropic or equivalent isotropic displacement parameters (Å^2^)

**Table:**

	*x*	*y*	*z*	*U*_iso_*/*U*_eq_	
C1	0.5938 (5)	0.3609 (4)	0.6172 (5)	0.0588 (17)	
C2	0.5803 (4)	0.2626 (4)	0.6825 (3)	0.0399 (11)	
H2	0.5450	0.2744	0.7426	0.048*	
C3	0.6209 (3)	0.1482 (3)	0.6545 (3)	0.0250 (9)	
C4	0.6703 (4)	0.1374 (4)	0.5656 (3)	0.0382 (11)	
H4	0.6988	0.0598	0.5462	0.046*	
C5	0.6797 (5)	0.2343 (6)	0.5047 (4)	0.0609 (16)	
H5	0.7123	0.2220	0.4436	0.073*	
C6	0.6442 (6)	0.3449 (6)	0.5291 (5)	0.0680 (18)	
H6	0.6532	0.4120	0.4869	0.082*	
C7	0.6123 (5)	0.0379 (4)	0.7189 (3)	0.0503 (13)	
H7A	0.6156	0.0645	0.7875	0.060*	
H7B	0.6865	−0.0156	0.7121	0.060*	
C8	0.4885 (4)	−0.0351 (4)	0.6931 (3)	0.0304 (10)	
H8A	0.4162	0.0131	0.7137	0.036*	
H8B	0.4758	−0.0436	0.6216	0.036*	
C9	0.5715 (4)	−0.2420 (4)	0.6958 (4)	0.0490 (13)	
H9A	0.6587	−0.2249	0.7233	0.074*	
H9B	0.5661	−0.2326	0.6250	0.074*	
H9C	0.5486	−0.3256	0.7119	0.074*	
C10	0.4948 (4)	−0.1550 (4)	0.8423 (3)	0.0348 (11)	
H10	0.5795	−0.1212	0.8675	0.042*	
C11	0.3871 (4)	−0.0811 (4)	0.8822 (3)	0.0408 (12)	
H11	0.3956	0.0094	0.8774	0.049*	
C12	0.3700 (5)	−0.1303 (4)	0.9854 (4)	0.0506 (13)	
H12	0.3736	−0.0702	1.0400	0.061*	
C13	0.4537 (4)	−0.2406 (5)	0.9980 (3)	0.0502 (14)	
H13	0.5343	−0.2286	1.0409	0.060*	
C14	0.4724 (4)	−0.2781 (4)	0.8898 (3)	0.0479 (13)	
H14	0.5445	−0.3364	0.8851	0.057*	
C15	0.3636 (5)	−0.3402 (5)	1.0322 (4)	0.0542 (14)	
H15A	0.4011	−0.4229	1.0328	0.065*	
H15B	0.3314	−0.3216	1.0957	0.065*	
C16	0.2635 (4)	−0.3182 (5)	0.9450 (3)	0.0470 (13)	
H16	0.1859	−0.3705	0.9451	0.056*	
C17	0.3392 (4)	−0.3332 (4)	0.8509 (4)	0.0469 (13)	
H17	0.3429	−0.4186	0.8262	0.056*	
C18	0.2639 (4)	−0.2484 (4)	0.7847 (3)	0.0379 (11)	
C19	0.2540 (4)	−0.1331 (5)	0.8456 (3)	0.0429 (12)	
H19	0.1893	−0.0720	0.8196	0.052*	
C20	0.2372 (4)	−0.1834 (4)	0.9485 (3)	0.0435 (12)	
H20	0.1614	−0.1557	0.9812	0.052*	
N1	0.4837 (3)	−0.1570 (3)	0.7364 (2)	0.0259 (8)	
O1	0.2010 (3)	−0.2730 (3)	0.70829 (19)	0.0431 (9)	
F1	0.5547 (4)	0.4710 (3)	0.6455 (4)	0.128 (2)	

### Atomic displacement parameters (Å^2^)

**Table:**

	*U*^11^	*U*^22^	*U*^33^	*U*^12^	*U*^13^	*U*^23^
C1	0.035 (3)	0.016 (2)	0.119 (5)	0.004 (2)	−0.030 (3)	−0.007 (3)
C2	0.023 (2)	0.053 (3)	0.043 (3)	−0.007 (2)	−0.0052 (18)	−0.016 (2)
C3	0.028 (2)	0.026 (2)	0.020 (2)	−0.0055 (18)	−0.0039 (16)	0.0031 (16)
C4	0.036 (2)	0.045 (3)	0.033 (3)	0.001 (2)	0.0019 (19)	−0.007 (2)
C5	0.044 (3)	0.104 (5)	0.034 (3)	−0.023 (3)	−0.001 (2)	0.017 (3)
C6	0.056 (4)	0.070 (4)	0.074 (4)	−0.032 (3)	−0.019 (3)	0.038 (4)
C7	0.053 (3)	0.047 (3)	0.046 (3)	−0.028 (2)	−0.021 (2)	0.021 (2)
C8	0.028 (2)	0.036 (2)	0.026 (2)	−0.0048 (19)	−0.0042 (17)	0.0024 (18)
C9	0.039 (3)	0.044 (3)	0.063 (3)	0.007 (2)	0.001 (2)	−0.011 (2)
C10	0.031 (2)	0.041 (3)	0.030 (2)	−0.017 (2)	−0.0098 (18)	0.0099 (19)
C11	0.046 (3)	0.050 (3)	0.027 (2)	−0.023 (2)	0.009 (2)	−0.006 (2)
C12	0.066 (3)	0.043 (3)	0.040 (3)	−0.015 (3)	−0.009 (2)	−0.005 (2)
C13	0.031 (2)	0.090 (4)	0.028 (2)	−0.011 (3)	−0.0097 (19)	0.012 (2)
C14	0.040 (3)	0.044 (3)	0.056 (3)	−0.014 (2)	−0.015 (2)	0.020 (2)
C15	0.057 (3)	0.059 (3)	0.045 (3)	−0.008 (3)	−0.006 (2)	0.018 (2)
C16	0.046 (3)	0.070 (4)	0.023 (2)	−0.028 (3)	−0.003 (2)	0.006 (2)
C17	0.042 (3)	0.037 (3)	0.058 (3)	−0.016 (2)	−0.013 (2)	0.010 (2)
C18	0.033 (2)	0.051 (3)	0.028 (2)	−0.019 (2)	−0.0031 (19)	0.008 (2)
C19	0.039 (3)	0.058 (3)	0.033 (3)	−0.006 (2)	0.005 (2)	0.001 (2)
C20	0.039 (3)	0.060 (3)	0.032 (3)	−0.002 (2)	0.006 (2)	0.008 (2)
N1	0.0248 (17)	0.0248 (18)	0.0278 (19)	−0.0006 (15)	0.0002 (13)	0.0012 (14)
O1	0.0350 (16)	0.070 (2)	0.0232 (16)	−0.0208 (16)	−0.0058 (13)	0.0024 (14)
F1	0.088 (3)	0.038 (2)	0.246 (6)	0.0133 (19)	−0.048 (3)	−0.037 (2)

### Geometric parameters (Å, °)

**Table:**

C1—F1	1.347 (6)	C10—H10	1.0000
C1—C6	1.384 (9)	C11—C12	1.551 (7)
C1—C2	1.422 (7)	C11—C19	1.557 (6)
C2—C3	1.393 (6)	C11—H11	1.0000
C2—H2	0.9500	C12—C13	1.499 (7)
C3—C4	1.382 (6)	C12—C20	1.560 (7)
C3—C7	1.511 (6)	C12—H12	1.0000
C4—C5	1.366 (7)	C13—C15	1.551 (7)
C4—H4	0.9500	C13—C14	1.581 (7)
C5—C6	1.323 (8)	C13—H13	1.0000
C5—H5	0.9500	C14—C17	1.579 (6)
C6—H6	0.9500	C14—H14	1.0000
C7—C8	1.546 (6)	C15—C16	1.546 (6)
C7—H7A	0.9900	C15—H15A	0.9900
C7—H7B	0.9900	C15—H15B	0.9900
C8—N1	1.469 (5)	C16—C20	1.508 (7)
C8—H8A	0.9900	C16—C17	1.595 (7)
C8—H8B	0.9900	C16—H16	1.0000
C9—N1	1.463 (5)	C17—C18	1.484 (6)
C9—H9A	0.9800	C17—H17	1.0000
C9—H9B	0.9800	C18—O1	1.225 (5)
C9—H9C	0.9800	C18—C19	1.529 (7)
C10—N1	1.458 (5)	C19—C20	1.550 (6)
C10—C14	1.531 (6)	C19—H19	1.0000
C10—C11	1.538 (6)	C20—H20	1.0000
			
F1—C1—C6	121.4 (6)	C13—C12—H12	117.7
F1—C1—C2	116.5 (6)	C11—C12—H12	117.7
C6—C1—C2	122.0 (5)	C20—C12—H12	117.7
C3—C2—C1	117.2 (4)	C12—C13—C15	103.6 (4)
C3—C2—H2	121.4	C12—C13—C14	102.9 (3)
C1—C2—H2	121.4	C15—C13—C14	103.7 (4)
C4—C3—C2	118.3 (4)	C12—C13—H13	115.0
C4—C3—C7	120.2 (4)	C15—C13—H13	115.0
C2—C3—C7	121.5 (4)	C14—C13—H13	115.0
C5—C4—C3	122.4 (5)	C10—C14—C17	111.0 (3)
C5—C4—H4	118.8	C10—C14—C13	102.3 (4)
C3—C4—H4	118.8	C17—C14—C13	103.8 (4)
C6—C5—C4	121.3 (5)	C10—C14—H14	113.0
C6—C5—H5	119.3	C17—C14—H14	113.0
C4—C5—H5	119.3	C13—C14—H14	113.0
C5—C6—C1	118.7 (5)	C16—C15—C13	92.6 (3)
C5—C6—H6	120.6	C16—C15—H15A	113.2
C1—C6—H6	120.6	C13—C15—H15A	113.2
C3—C7—C8	112.0 (3)	C16—C15—H15B	113.2
C3—C7—H7A	109.2	C13—C15—H15B	113.2
C8—C7—H7A	109.2	H15A—C15—H15B	110.5
C3—C7—H7B	109.2	C20—C16—C15	104.1 (4)
C8—C7—H7B	109.2	C20—C16—C17	103.6 (4)
H7A—C7—H7B	107.9	C15—C16—C17	105.2 (4)
N1—C8—C7	116.0 (3)	C20—C16—H16	114.2
N1—C8—H8A	108.3	C15—C16—H16	114.2
C7—C8—H8A	108.3	C17—C16—H16	114.2
N1—C8—H8B	108.3	C18—C17—C14	112.3 (3)
C7—C8—H8B	108.3	C18—C17—C16	99.1 (4)
H8A—C8—H8B	107.4	C14—C17—C16	100.2 (4)
N1—C9—H9A	109.5	C18—C17—H17	114.4
N1—C9—H9B	109.5	C14—C17—H17	114.4
H9A—C9—H9B	109.5	C16—C17—H17	114.4
N1—C9—H9C	109.5	O1—C18—C17	127.6 (4)
H9A—C9—H9C	109.5	O1—C18—C19	126.7 (4)
H9B—C9—H9C	109.5	C17—C18—C19	103.9 (4)
N1—C10—C14	114.6 (3)	C18—C19—C20	103.2 (4)
N1—C10—C11	112.0 (3)	C18—C19—C11	112.2 (4)
C14—C10—C11	99.4 (3)	C20—C19—C11	90.4 (3)
N1—C10—H10	110.1	C18—C19—H19	115.9
C14—C10—H10	110.1	C20—C19—H19	115.9
C11—C10—H10	110.1	C11—C19—H19	115.9
C10—C11—C12	107.3 (4)	C16—C20—C19	106.5 (4)
C10—C11—C19	111.3 (3)	C16—C20—C12	102.4 (4)
C12—C11—C19	89.7 (3)	C19—C20—C12	89.6 (3)
C10—C11—H11	115.2	C16—C20—H20	118.0
C12—C11—H11	115.2	C19—C20—H20	118.0
C19—C11—H11	115.2	C12—C20—H20	118.0
C13—C12—C11	105.8 (4)	C10—N1—C9	113.5 (3)
C13—C12—C20	103.8 (4)	C10—N1—C8	113.1 (3)
C11—C12—C20	90.3 (3)	C9—N1—C8	112.3 (3)
			
F1—C1—C2—C3	−179.7 (4)	C13—C14—C17—C18	104.4 (4)
C6—C1—C2—C3	0.5 (6)	C10—C14—C17—C16	−109.2 (4)
C1—C2—C3—C4	−0.6 (6)	C13—C14—C17—C16	0.0 (4)
C1—C2—C3—C7	179.2 (4)	C20—C16—C17—C18	−41.0 (4)
C2—C3—C4—C5	−0.4 (6)	C15—C16—C17—C18	−150.0 (4)
C7—C3—C4—C5	179.8 (4)	C20—C16—C17—C14	73.7 (4)
C3—C4—C5—C6	1.6 (7)	C15—C16—C17—C14	−35.3 (5)
C4—C5—C6—C1	−1.6 (8)	C14—C17—C18—O1	137.7 (5)
F1—C1—C6—C5	−179.2 (5)	C16—C17—C18—O1	−117.2 (5)
C2—C1—C6—C5	0.6 (8)	C14—C17—C18—C19	−56.7 (5)
C4—C3—C7—C8	−85.8 (5)	C16—C17—C18—C19	48.4 (4)
C2—C3—C7—C8	94.4 (5)	O1—C18—C19—C20	127.7 (4)
C3—C7—C8—N1	167.1 (4)	C17—C18—C19—C20	−38.0 (4)
N1—C10—C11—C12	154.8 (3)	O1—C18—C19—C11	−136.4 (4)
C14—C10—C11—C12	33.3 (4)	C17—C18—C19—C11	58.0 (4)
N1—C10—C11—C19	58.2 (4)	C10—C11—C19—C18	3.5 (5)
C14—C10—C11—C19	−63.3 (4)	C12—C11—C19—C18	−105.0 (4)
C10—C11—C12—C13	−7.4 (5)	C10—C11—C19—C20	108.1 (4)
C19—C11—C12—C13	104.9 (4)	C12—C11—C19—C20	−0.5 (4)
C10—C11—C12—C20	−111.8 (4)	C15—C16—C20—C19	128.1 (4)
C19—C11—C12—C20	0.5 (4)	C17—C16—C20—C19	18.3 (4)
C11—C12—C13—C15	−128.9 (4)	C15—C16—C20—C12	34.8 (4)
C20—C12—C13—C15	−34.7 (4)	C17—C16—C20—C12	−75.0 (4)
C11—C12—C13—C14	−21.1 (4)	C18—C19—C20—C16	10.6 (4)
C20—C12—C13—C14	73.1 (4)	C11—C19—C20—C16	−102.3 (4)
N1—C10—C14—C17	−55.1 (5)	C18—C19—C20—C12	113.4 (4)
C11—C10—C14—C17	64.4 (5)	C11—C19—C20—C12	0.5 (4)
N1—C10—C14—C13	−165.3 (3)	C13—C12—C20—C16	0.0 (4)
C11—C10—C14—C13	−45.8 (4)	C11—C12—C20—C16	106.3 (4)
C12—C13—C14—C10	42.8 (4)	C13—C12—C20—C19	−106.8 (4)
C15—C13—C14—C10	150.5 (4)	C11—C12—C20—C19	−0.5 (4)
C12—C13—C14—C17	−72.8 (4)	C14—C10—N1—C9	−58.3 (5)
C15—C13—C14—C17	34.9 (5)	C11—C10—N1—C9	−170.6 (3)
C12—C13—C15—C16	53.3 (4)	C14—C10—N1—C8	172.2 (3)
C14—C13—C15—C16	−53.9 (4)	C11—C10—N1—C8	59.9 (4)
C13—C15—C16—C20	−53.6 (4)	C7—C8—N1—C10	61.1 (5)
C13—C15—C16—C17	55.0 (4)	C7—C8—N1—C9	−69.0 (5)
C10—C14—C17—C18	−4.9 (6)		
